# A virtual surgical prototype system based on gesture recognition for virtual surgical training in maxillofacial surgery

**DOI:** 10.1007/s11548-022-02790-1

**Published:** 2022-11-23

**Authors:** Hanjiang Zhao, Mengjia Cheng, Jingyang Huang, Meng Li, Huanchong Cheng, Kun Tian, Hongbo Yu

**Affiliations:** 1 Department of Oral and Cranio-Maxillofacial Surgery, Shanghai Ninth People’s Hospital, Shanghai Jiao Tong University School of Medicine; College of Stomatology, Shanghai Jiao Tong University; National Center for Stomatology; National Clinical Research Center for Oral Diseases; Shanghai Key Laboratory of Stomatology, Shanghai Research Institute of Stomatology, No. 639 Zhizaoju Road, Shanghai, 200011 China; 2 Department of Stomatology, The First Hospital of Zibo, Zibo, 255200 Shandong Province China; 3 School of Mechanical and Power Engineering, Shanghai Jiao Tong University, No. 800 Dongchuan Road, Shanghai, 200240 China

**Keywords:** Virtual reality, Gesture recognition, Collision detection, Virtual surgery

## Abstract

**Background:**

Virtual reality (VR) technology is an ideal alternative of operation training and surgical teaching. However, virtual surgery is usually carried out using the mouse or data gloves, which affects the authenticity of virtual operation. A virtual surgery system with gesture recognition and real-time image feedback was explored to realize more authentic immersion.

**Method:**

Gesture recognition technology proposed with an efficient and real-time algorithm and high fidelity was explored. The recognition of hand contour, palm and fingertip was firstly realized by hand data extraction. Then, an Support Vector Machine classifier was utilized to classify and recognize common gestures after extraction of feature recognition. The algorithm of collision detection adopted Axis Aligned Bounding Box binary tree to build hand and scalpel collision models. What’s more, nominal radius theorem (NRT) and separating axis theorem (SAT) were applied for speeding up collision detection. Based on the maxillofacial virtual surgical system we proposed before, the feasibility of integration of the above technologies in this prototype system was evaluated.

**Results:**

Ten kinds of signal static gestures were designed to test gesture recognition algorithms. The accuracy of gestures recognition is more than 80%, some of which were over 90%. The generation speed of collision detection model met the software requirements with the method of NRT and SAT. The response time of gesture] recognition was less than 40 ms, namely the speed of hand gesture recognition system was greater than 25 Hz. On the condition of integration of hand gesture recognition, typical virtual surgical procedures including grabbing a scalpel, puncture site selection, virtual puncture operation and incision were carried out with realization of real-time image feedback.

**Conclusion:**

Based on the previous maxillofacial virtual surgical system that consisted of VR, triangular mesh collision detection and maxillofacial biomechanical model construction, the integration of hand gesture recognition was a feasible method to improve the interactivity and immersion of virtual surgical operation training.

## Introduction

With the development of digital technology, computer-aided surgery has received a wide application [1, 2]. As the most developed and validated of the reality technology, virtual reality (VR) technology can provide us an interactive practice environment for surgical teaching and operation training [3]. It is a simulation system using computer to generate and explore a visual simulation environment with multi-source information fusion, interactive three-dimensional dynamic view and entity behavior [4].Currently, some of available VR training systems emerge on the market for orthopedic surgery with haptic device technology, which allow trainees feel the operation of virtual surgical instruments on bones by transmitting force feedback onto the user's real hands [5].

In order to fulfill natural physician–computer interaction, gesture recognition, the technology that recognizes the hand gestures of users directly in the virtual environment and generates various image feedbacks according to the gestures [6–8], has great potential in the field of medicine including rehabilitation, imageology and education [9–11]. In virtual surgery, the collision detection (CD) is indispensable, which used to detect whether there is overlap between one or more geometric objects in space [12]. The contact among the virtual hand, scalpel and tissue needs to be explored.

Although existing VR-related surgery training system have significantly improved the authenticity of anatomical structures and morphology, the realization of human touch that comes with visual and haptic feedback and construction of physical model based on anatomical human tissue characteristics, especially soft tissue, is still the challenge for surgical simulation [13, 14]. On the other hand, the input equipment such like mouse or digital gloves is required as indirect man–machine interaction tools during virtual operation, which is different from the real surgery performed by hands grasping surgical instrument [15–18]. What’s more, the combined application in maxillofacial preoperative design, operation training and surgical education was rarely reported [19].

As a basic operation, facial soft tissue incision is widely used for most of maxillofacial surgeries including various extraoral incisions such as preauricular incision for temporomandibular joint related surgery. According to our previous work, the virtual surgical prototype system was constructed with the maxillofacial soft tissue biomechanical model, which could plan the path and length of incisions, detect the bone surface and stop moving deeper and set facial soft tissue as semitransparent display mode [20]. However, the interactivity and immersion during virtual operation were still not satisfying. To further improve the effect of virtual surgery, a modified maxillofacial virtual surgery training system combined with gesture recognition, soft tissue biomechanical model and collision detection is explored, which is of great significance to the cultivation of oral and maxillofacial surgeons.

## Materials and methods

### Gesture recognition

**Fig. 1:**
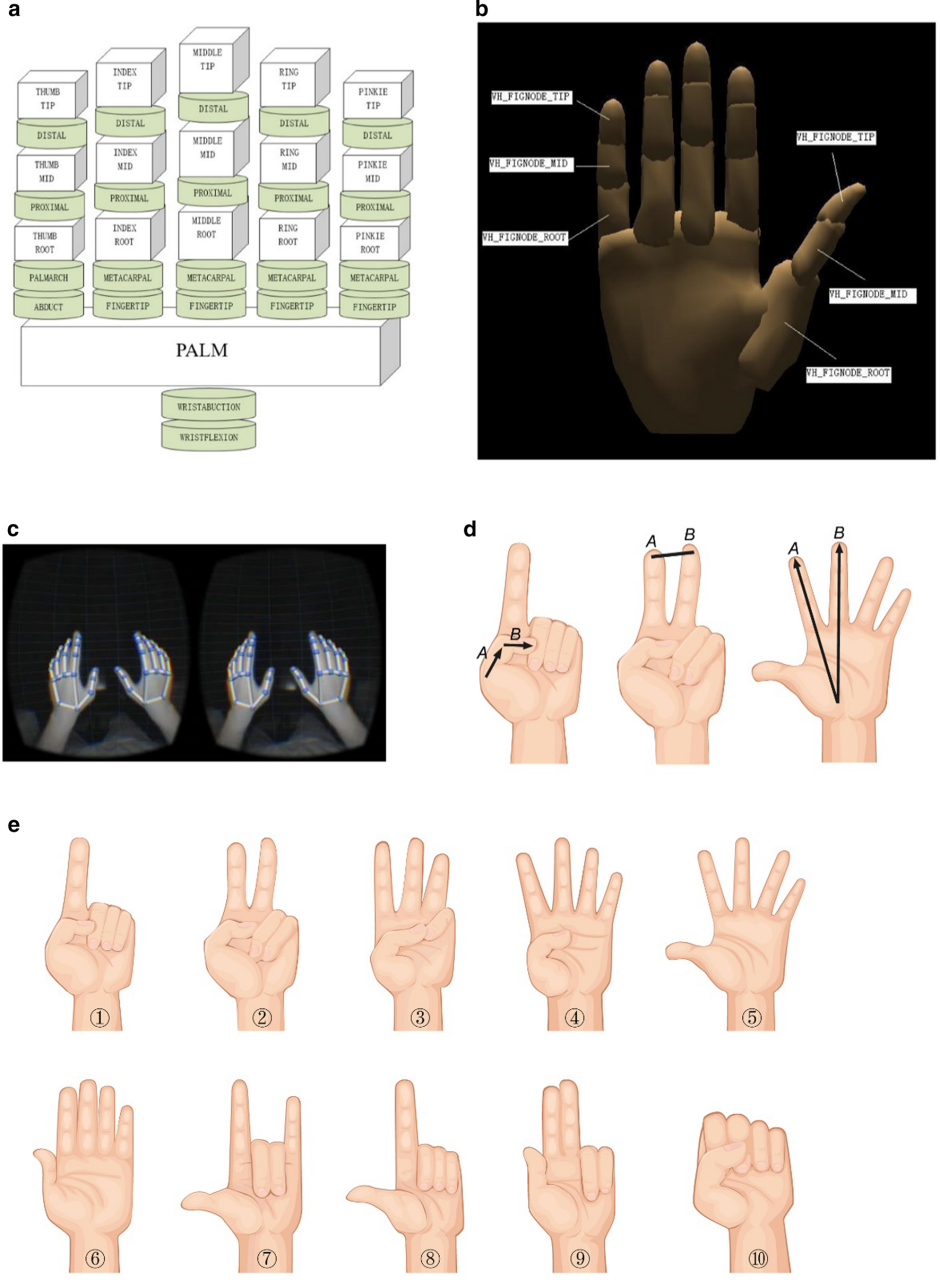
The construction of virtual hand model and hand gesture recognition. **A** The virtual hand model component. **B** The structure of virtual hand model. **C** The actual hand posture acquisition in virtual space. **D** The hand curl, number of outstretched fingers, angle and distance between adjacent fingers for SVM (Support Vector Machine) classifier establishment. **E** 10 kinds of signal static hand gestures for testing

According to the anatomical structure and motion characteristics of hand, a virtual hand model with 15 rigid bodies and 22 degrees of freedom was established to express the geometric and kinematic characteristics (Fig. 1A). As the smallest component of the virtual hand, the virtual hand model component was considered as a rigid body during movement, corresponding to the anatomical structure of bones in hand. Virtual hand joints were utilized to connect each component, and the motion constraints between components were realized by cylindrical hinge constraints (Fig. 1B). Virtual hand action events were used to trigger the instantaneous events of virtual hand state changes, controlling the current spatial position, geometric posture and grasping state of the virtual hand.

The Microsoft’s Kinect™ (Kinect One) was used to track fingers in a way based on the time of flight (TOF) technology, which is different from common motion control technologies. The round-trip time of the light is measured to determine the distance, which meant the depth of the whole scene was continuously transmitted and captured by modulating the light pulse during the scan. The same scene was shot by two cameras in different positions on the same plane, and then the parallax of space points in two images was calculated by triangulation method, so as to calculate the position of the target in three-dimensional space. The distance between hand and Kinect One during experiments was less than one meter.

Combined with the virtual hand model, the spatial posture transformation matrix of the virtual hand was established through six degrees of freedom of the palm and 22 degrees of freedom of the virtual hand. The matrix was solved according to the contour position and depth information obtained from the image recognition, and the actual posture of the virtual hand in space was obtained (Fig. 1c).

An SVM (Support Vector Machine) classifier was established based on the feature set of gesture recognition including hand curl, number of outstretched fingers, angle and distance between adjacent fingers. The optimal classification function derived for the linear separable problem can be formulated as follows:

$$f(x)=\upvarepsilon \left\{({w}^{*}\cdot x)+{b}^{*}\right\}=\varepsilon \left\{\sum\limits_{i=1}^{n}{a}_{i}^{*}{y}_{i}({x}_{i}\cdot x)+{b}^{*}\right\}$$

For linear inseparable problems, penalty term C and slack variables can be introduced to solve them. However, SVM algorithm needs to map the features of low-dimensional samples in the feature space of high-dimensional samples to solve nonlinear problems, and then transform the nonlinear separable case into the linear separable case. Therefore, kernel function *K*(*x*_*i*_*,* *x*_*j*_) is introduced to deal with this problem. It can be obtained by introducing kernel function into discriminant function as follows:

$$f(x)=\varepsilon \left\{\sum\limits_{i=1}^{n}{a}_{i}^{*}{{x}_{i}K(x}_{i}\cdot {x}_{j})+{b}^{*}\right\}$$

RBF (radial basis function) kernel function has certain locality and sample data can be mapped in higher dimensions. However, the generalization ability is slightly weak, and it is negatively correlated with the parameter σ [21]:

$${K(x}_{i}\cdot {x}_{j})=\mathrm{exp}\left(-\frac{{\Vert {x}_{i}-{x}_{j}\Vert }^{2}}{2{\sigma }^{2}}\right)$$

In addition to determining the parameters of kernel function, penalty term *C* will affect its complexity and approach error, while parameter *γ* is used to control the error value of regression approximation. Besides, the OVO (One Versus One) strategy classified into multi-class classification problems (Fig. 1D). 10 kinds of signal static hand gestures were selected as recognition objects according to the recognition speed and robustness (Fig. 1E). Finally, the SVM module of Scikit–Lean and the RBF kernel function were used to train the multi-class SVM classifier.

250 sets of data samples from 5 testers were collected for training, validation and testing. These five testers consisting of 3 males and 2 females were surgeons in the Department of Oral and Craniomaxillofacial Surgery between the ages of 27 and 35. The data samples of each hand gesture were divided into training, validation and test set in a ratio of 3:1:1. The method used for parameter selection was fourfold cross-validation. At each partition of the 200 sets of data samples, training and validation are performed on different datasets to obtain evaluation results. Finally, the evaluation results after four divisions are obtained and the final score is obtained by averaging the evaluation results of these four times.

### Collision model construction and collision detection algorithm

**Fig. 2:**
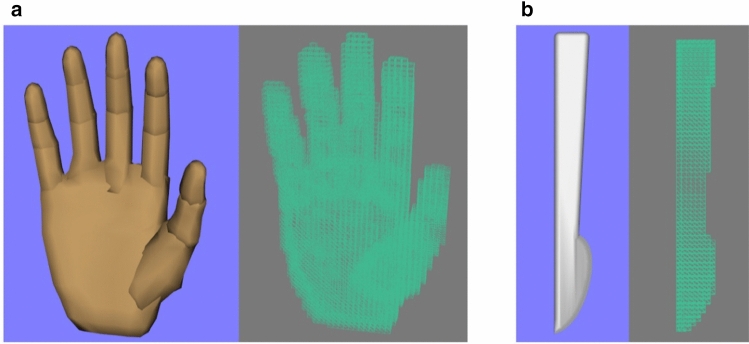
The reconstruction of virtual hand and scalpel collision model comprised of triangular facets

Similar with soft tissue collision model described in our previous study [20], the AABB binary tree was conducted to organize the collision model, which contains description information, facet information (point, normal vector) and bounding box binary tree information. The virtual hand and scalpel models were converted into collision models comprised of triangular facets in the virtual surgical system (Fig. 2A, B). The whole virtual hand and scalpel model consisted of 3538 and 2336 triangle elements respectively.

**Fig. 3:**
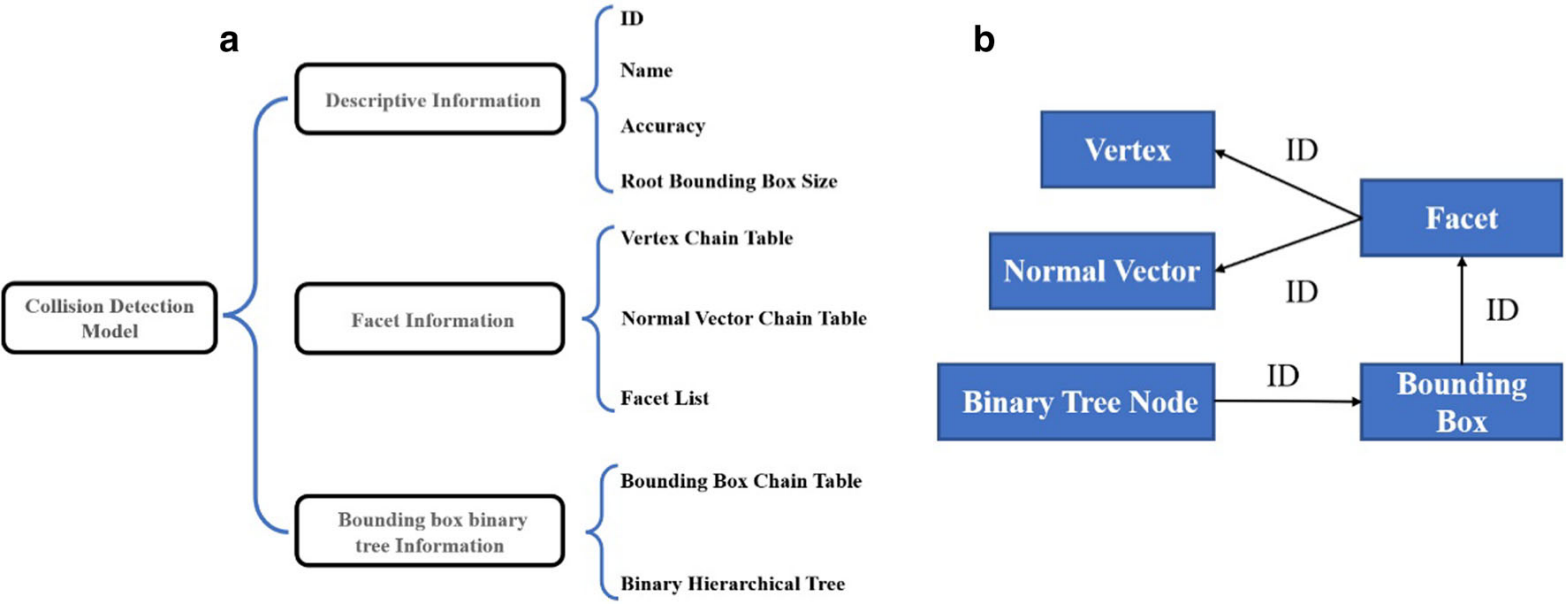
Collision detection model based on AABB bounding box binary tree. **A** The organization structure of collision detection model. **B** The inner relationship among binary tree node, bounding box, facet, vertex and normal vector indexed by ID

Among them, description information referred to the basic information of the model, such as model ID, name, accuracy and size of root bounding box, and etc. Facet information included vertex linked list, normal vector linked list and facet list, which was mainly used for precise collision detection. Bounding box binary tree information included bounding box linked list (storing bounding box data distributed in different levels) and binary hierarchical tree (storing binary tree nodes, including one parent node and two child nodes), which was applied to collision detection based on bounding box (Fig. 3A). It was worth noting that binary tree nodes correspond to bounding boxes one by one, searching each other by ID. A bounding box contained a number of facets and is indexed by facets ID, while a facet consisted of several vertices and a normal vector, which are indexed by vertex ID and normal vector ID (Fig. 3B).

Collision detection between bounding levels was firstly judged by nominal radius theorem (NRT) to achieve fast implementation of detection. The intersection detection between bounding box and facet was the main link that affects the generation speed of collision detection model. Since the actual surface was usually triangular, the project adopted the separating axis theorem (SAT) to accelerate the detection speed under the circumstances that it was unable to judge by NRT.

### Virtual maxillofacial surgical prototype system development

Based on maxillofacial biomechanical model and collision detection mentioned in previous research [20], virtual reality (VR) technology was applied to develop a prototype maxillofacial virtual surgery system supporting gesture recognition, which could perform interactive maxillofacial surgery in three-dimensional (3D) environment. Among them, collision detection was pure CPU computation, gesture recognition could be accelerated by GPU, and graphics rendering was pure GPU operation. The system was developed by C++ language to simulate the basic operation of maxillofacial tissue surgery in a 3D environment.

**Fig. 4:**
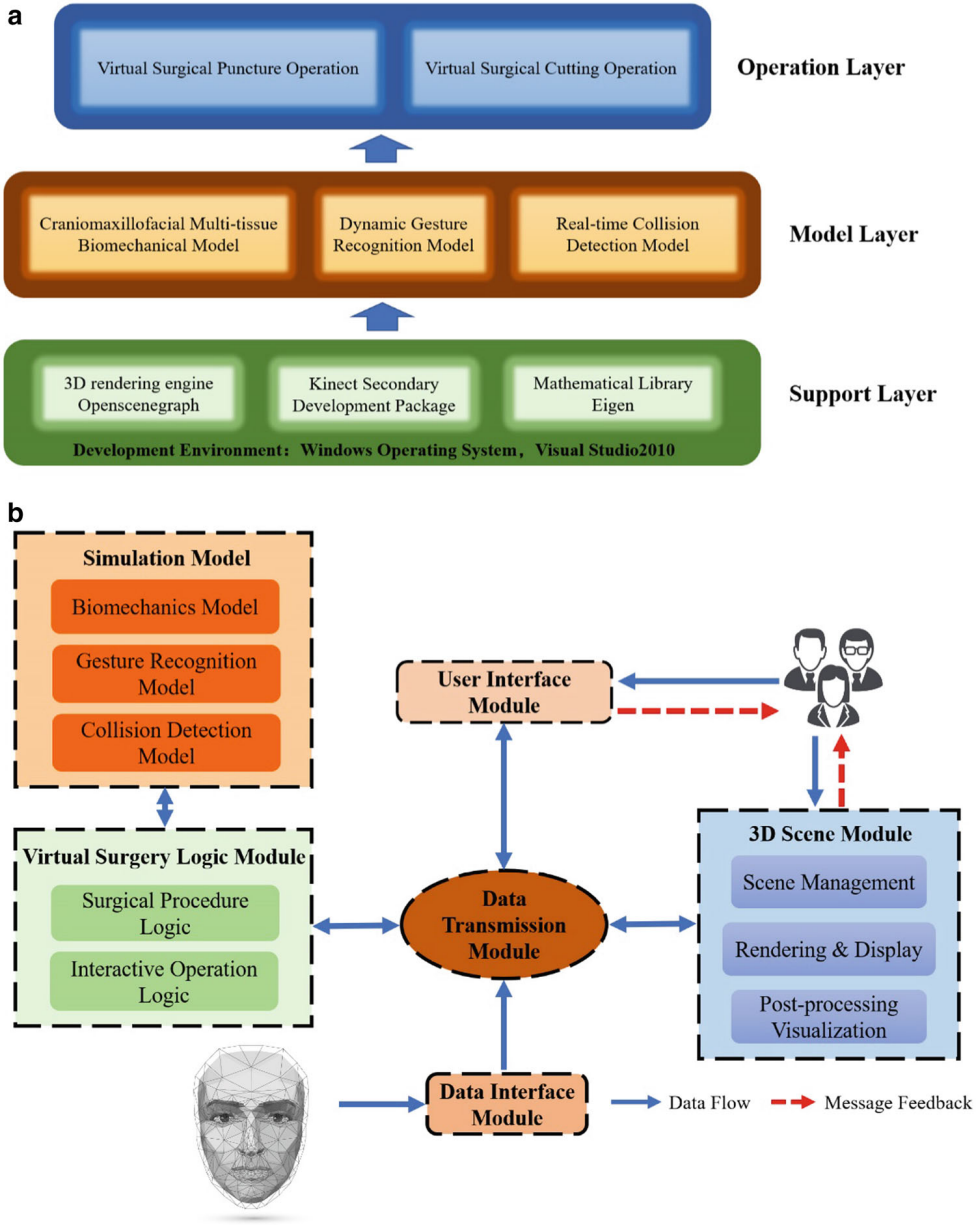
The prototype system hierarchy structure and system component module for maxillofacial virtual surgery supporting gesture recognition

The system hierarchy structure was divided into three levels, which contained support layer, model layer and operation layer from bottom to top (Fig. 4A). Support layer was the bottom support toolkit and development library of the system including Openscenegraph library, graphics rendering library that supported 3D scene display, a secondary development package that supported Kinect data collection and Eigen, a mathematics library that supported matrix operation, etc. According to the key technology of this research, model layer involved maxillofacial multi-tissue biomechanical model supporting soft tissue deformation, dynamic gesture recognition model and real-time collision detection model. On the basis of model layer, the virtual operation logic model of maxillofacial tissue was realized, including two main functions: virtual surgical insertion and cutting operation.

The prototype system of craniomaxillofacial virtual surgery supporting gesture recognition adopted modular design including user interface module, virtual surgery logic module, 3D scene module, simulation support module, data transmission module and data interface module (Fig. 4B).

Among them, the simulation support module contained the key technologies in this study, including maxillofacial tissue biomechanics model, gesture recognition model and collision detection model, and etc. This module only transmitted data as a support tool for other modules rather than operates independently.

The virtual operation process of maxillofacial surgery was controlled and interacted by virtual surgical logic module that can mobilize each model in the simulation support module to realize the main functions of virtual and real surgery.

The user interface module mainly contained the menu, function dialog box, shortcut function keys, and etc., which was mainly used for mode selection, parameters input, parameters check, and etc. realized by the two-dimensional menu from the interface to achieve human–computer interaction operation function.

The 3D scene module mainly contained scene management, rendering and display functions, as well as post-processing visualization functions for soft tissue deformation calculation, supporting interactive operations such as viewpoint movement and object picking in the scene and realizing human–computer interaction in 3D environment by rendering the images obtained from the scene.

The data transmission module was applied on message encoding, decoding and data transmission among other modules. Each module has established a message receiving and sending cache queue and asynchronous message transmission, supporting memory data transmission, disk file transmission and so on.

The data interface module realized the transformation function of 3D data, converting 3D soft tissue model from CT, scalpel 3D model and virtual hand 3D model scan into 3D data format that can be recognized by this system.

## Results

### Gesture recognition test

**Fig. 5:**
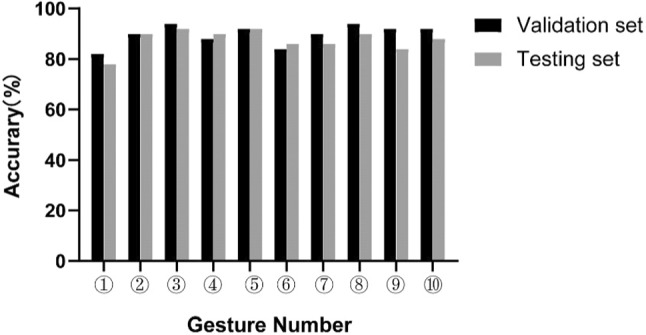
The accuracy of hand gesture recognition for validation and testing set

**Fig. 6:**
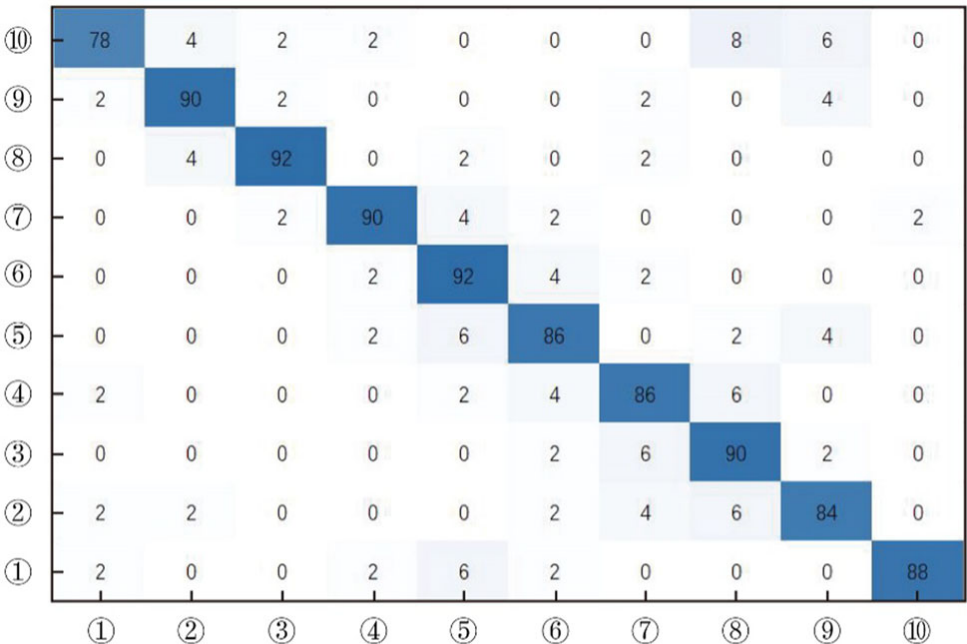
The confusion matrix of the proposed approach for testing set

According to the recognition speed and robustness, 10 kinds of signal static gestures were selected as recognition objects, which were easy to show and had obvious features. At the training stage, the online recognition stage was carried out, which required fast recognition speed and high accuracy. The obtained gesture feature of known categories was constructed as a column vector. Input the column vector and corresponding labels into Scikit–Lean, and the labels were arranged in sequence to map to various gestures. The parameters of the training set were used to find the optimal parameters of the data set and then the model parameters were further adjusted by the validation set. The results show that when C = 0.8, γ = 3, the recognition result of the training set is optimal. As shown in the Fig. 5, all gestures achieved satisfied recognition accuracy with more than 80%. Besides, there was no significant difference in the accuracy of gesture recognition between training samples and test samples. The confusion matrix of the proposed approach for testing set was shown in Fig. 6.

### Virtual maxillofacial surgery

According to our previous study, the biomechanical parameters of facial soft tissue model was utilized as the object of virtual maxillofacial surgery prototype system integrated with gesture recognition and collision detection modules. The typical virtual operation process on soft tissue included three main steps: puncture site selection, virtual puncture and virtual incision operation. In each step, the hand posture obtained by gesture recognition module was used as input. After each step was completed, the "Next step" gesture was directly used to enter the next step.

**Fig. 7:**
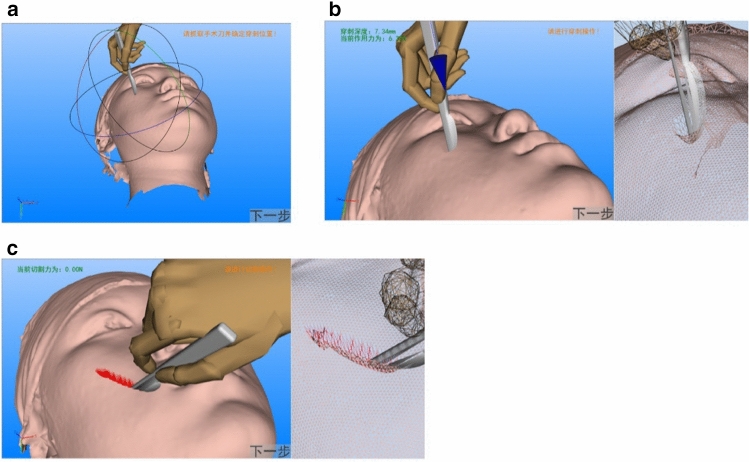
The procedure of virtual operation. **A** The step of virtual puncture point selection. **B** The step of virtual puncture operation. **C** The step of Virtual cutting operation

The virtual surgery system could be started by hand gesture No.5 and ended by hand gesture No.10. Firstly, the virtual scalpel was captured in the virtual environment. Then, the puncture point was determined on maxillofacial soft tissue (Fig. 7A). The system captured the user's hand position through Kinect 3D camera, acquiring the angle values of each joint of the real hand in real time and synchronizing the posture of the virtual hand in the virtual surgical environment. It was remarkable that the system detected the position relationship between the virtual hand and the virtual scalpel through the collision detection function. When the virtual hand touched the virtual scalpel, the grasping operation performed to associate the position of the scalpel and hand. After the capture, the user moved the scalpel through interactive operation, the system detected the collision relationship between the virtual scalpel and soft tissue. When collision occurred, the collision point was used as the puncture point. The user confirmed the puncture point by gesture to conform the virtual puncture operation.

At the selected puncture point, the virtual scalpel was controlled by recognition to carry out puncture operation (Fig. 7B). In the frame loop, the system obtained the penetrating depth of the scalpel relative to the soft tissue model through collision detection. The displacement of penetrating depth was taken as the input of the biomechanical model of maxillofacial soft tissue to calculate the deformation of the entire triangular mesh. When scalpel reached a certain depth, the triangular mesh model was destroyed and the soft tissue model was cut in a neat line.

When puncture operation finished, the virtual scalpel was used for cutting operation (Fig. 7C). In this procedure, the system in the frame cycle updated the location information of scalpel based on identifying the virtual hand position and calculated length of incision according to the real-time scalpel position. Besides, a series of cascading program occurred on after another including the damage of triangular mesh during updated collision detection, the removal of the constrained relationship among triangular mesh and the reconstruction of triangular mesh model. At the same time, the color and texture of the triangular mesh model were modified in the visual rendering module to make the incision more consistent with real situation. When the operations completed, the virtual surgical process can be ended by hand gesture.

Previous work showed that the average 3D scene update rate of the system was 59.3 FPS (frames per second). The simulation calculation update rate was slightly slower (about 20–50 Hz). As an individual update thread, the process of gesture recognition didn’t affect the response time of the previous system. The average processing time of hand detection and tracking, image preprocessing and SVM classifier for gesture recognition were about 5 ms, 8 ms and 25 ms respectively. Taken together, the response time of gesture recognition was less than 40 ms, namely the speed of hand gesture recognition system was greater than 25 Hz.

The virtual surgical prototype system realized virtual incision on soft tissue combined with the application of gesture recognition technology, maxillofacial biomechanical deformable model and triangular mesh collision detection technology. This study verified the feasibility of gesture recognition integrated into virtual surgery simulation.

## Discussion

The training of surgeons requires a lot of time and clinical operation opportunities. Reducing costs of surgical training and decreasing training hours can be regarded as the goals of practical operation education [22, 23]. Compared with traditional surgical training, virtual surgery system constructs an authentic and highly interactive virtual environment to enhance the efficiency and effectiveness of surgery learning and training [1, 24]. With the introduction of collision detection, real-time image and haptic feedback, the immersion of VR-based virtual surgery system has been further improved [12–14]. Previous studies have confirmed that VR-based simulators with or without force feedback support effective learning reified as faster completion of surgery and less injury [20, 25–27]. However, the realism of the virtual surgery system from the real-time visual and tactile senses are still limited by the algorithms and equipment. The challenge of virtual surgery system was to integrate biomechanical model including both soft and hard tissue in region for haptic feedback sensations along with the combination of gesture recognition for better interaction.

At present time, gesture recognition becomes more and more widespread in medicine [28–30]. The majority of those works are adopted for contactless medical images visualization [31–33], presurgical diagnosis [34], virtual surgery design [35] and surgical robot training [36, 37]. As an alternative of human–computer interaction, gesture recognition with real-time graphical feedback and deformation also provides great promise in virtual surgery training [38]. Replacing the traditional operation mode with external device, gesture recognition was used to realize visually realistic surgical operation on maxillofacial multi-tissue model with hands and instruments in virtual environment.

Including SVM classifier we utilized in this work, K-Nearest Neighbor (KNN) and Random Forest (RF) were both classic machine learning classification algorithm. Based on statistics, KNN selected the largest number of samples from k nearest neighbor samples in the experimental samples and then classified them according to their characteristics. However, this simple method couldn’t handle samples with high dimensionality. Besides, compared with SVM classifier, KNN needed more calculation but the accuracy was relatively low. While RF owned great performance in machine learning, which could handle large datasets with large feature space and could be parallelized for both rapid training and recall performance [39, 40]. As for the applicaition in gesture recognition, Pugeault and Bowden adopted Random Forest to classify hand gestures corresponding to letters of the alphabet for efficient writing [39]. Dong et al. applied Random Forest classifier to recognize ASL signs using hand joint angles and acquired satisfying accuracy [41]. As for SVM classifier, according to previous study, Aamrah Ikram et al. proposed a new approach for efficient hand gesture recognition using Convolutional Neural Network (CNN) along with Support Vector Machine (SVM) classifier [42]. CNN was implemented to avoid feature extraction and to minimized the number of trained parameters while SVM classifier was used for system's validity and robustness. The results showed that the accuracy of the model was significantly enhanced by implementing CNN-SVM approach compared with CNN. Besides, considering the hand gestures during operation were relatively fixed, we chose SVM classifier to obtain satisfying recognition accuracy.

In our previous research, we built a maxillofacial virtual surgical system that enabled operators to simulate insertion and cutting on facial soft tissue based on maxillofacial physical model with realization of realistic deformation and haptic feedback [20]. Force Dimension’s omega 6 was used for haptic feedback and mouse was utilized for systematic operation such as the movement of model in three dimensions. To further improve the operational interactivity and surgical immersion, the gesture recognition technology was recommended for integration into this system. The significance of this during actual application scenarios was that: (1) The hand gestures could replace mouse and keyboard to complete some normal operation of the virtual surgical system such as movement, rotation and zooming in or out of 3D maxillofacial models. (2) Some special surgical hand gestures like holding a scalpel could be recognized whether it was correct, which was a natural way to use the apparatus. (3) As mentioned in previous study [43], the hand motion record and analysis of surgical trainees could be utilized for their skill assessment. Thus, conform to the trend of natural human–computer interaction moving from "computer-centric" to "human-centered", the research content of user experience in communicating with computer using simple and convenient gestures was profound and significant.

In this study, hand movements are recognized by the binary image of hand and depth value. Then, the main features of gestures are extracted by feature recognition technology, and common gestures are classified by SVM classifier. Finally, this gesture recognition module along with related collision detection technology has been added to our virtual surgical prototype system for further enhancement of the immersion of VR-based virtual surgery. What needed to be emphasized was that the gesture recognition used individual update thread, which meant gesture recognition didn’t affect the response time of the previous system.

The innovation of this prototype system was that gesture recognition technology proposed with an efficient and real-time algorithm and high fidelity was applied in maxillofacial virtual surgery. Besides, collision detection not only enhanced visual effects but also lay the foundation for tactile feedback. The BVTree technology greatly reduces the time complexity at the cost of slightly increasing the spatial complexity resulting from establishing outside layered bounding boxes. AABB Tree, the first layered bounding boxes, is the smallest hexahedron parallel to the coordinate axis. Therefore, these technologies based on our original algorithm have great value for visual feedback of virtual maxillofacial surgery. It provided an ideal alternative with extremely high economic value and social benefits for virtual surgical design, surgeon training and surgical simulation.

## Conclusion and further perspective

As a method of human–computer interaction, the integration of hand gesture recognition with high accuracy and effectiveness was a feasible method to improve the interactivity and immersion of virtual surgical operation training on the basis of previous virtual surgical system that combined with VR, triangular mesh collision detection and maxillofacial biomechanical model construction.

In the future, the recognition accuracy of the proposed approach will be optimized and more efficient algorithms for hand gesture recognition will be explored. More operations such as the movement of maxillofacial models in virtual surgery system need to be controlled by some specific hand gestures. Besides, if the biomechanical parameters operation of maxillofacial bones was integrated into the system as well, the function of this software will be applied for other surgical method such as orthognathic surgery. Furthermore, in order to further improve the authenticity of virtual surgery, some input devices shaped like scalpel, tweezer, vascular clamp and so on can be well-designed for specific application [44, 45]. With the continuous upgrade of hardware, the advanced equipment for augmented reality or mixed reality like Microsoft HoloLens with gesture tracking will further improve the effect of virtual surgery [46–48].

## References

[1] Mao RQ, Lan L, Kay J, Lohre R, Ayeni OR, Goel DP, Sa D (2021). Immersive virtual reality for surgical training: a systematic review. J Surg Res.

[2] Bartlett JD, Lawrence JE, Stewart ME, Nakano N, Khanduja V (2018). Does virtual reality simulation have a role in training trauma and orthopaedic surgeons?. Bone Joint J.

[3] Lungu AJ, Swinkels W, Claesen L, Tu P, Egger J, Chen X (2021). A review on the applications of virtual reality, augmented reality and mixed reality in surgical simulation: an extension to different kinds of surgery. Expert Rev Med Devices.

[4] Chou B, Handa VL (2006). Simulators and virtual reality in surgical education. Obstet Gynecol Clin North Am.

[5] Verhey JT, Haglin JM, Verhey EM, Hartigan DE (2020). Virtual, augmented, and mixed reality applications in orthopedic surgery. Int J Med Robot.

[6] Kim H, Kim Y, Lee EC (2014). Method for user interface of large displays using arm pointing and finger counting gesture recognition. Sci World J.

[7] Oudah M, Al-Naji A, Chahl J (2020). Hand gesture recognition based on computer vision: a review of techniques. J Imaging.

[8] Wei S, Chen X, Yang X, Cao S, Zhang X (2016). A component-based vocabulary-extensible sign language gesture recognition framework. Sensors (Basel).

[9] Mewes A, Hensen B, Wacker F, Hansen C (2017). Touchless interaction with software in interventional radiology and surgery: a systematic literature review. Int J Comput Assist Radiol Surg.

[10] Ponce BA, Menendez ME, Oladeji LO, Fryberger CT, Dantuluri PK (2014). Emerging technology in surgical education: combining real-time augmented reality and wearable computing devices. Orthopedics.

[11] Zhang J, Nie Y, Lyu Y, Yang X, Chang J, Zhang JJ (2021). SD-Net: joint surgical gesture recognition and skill assessment. Int J Comput Assist Radiol Surg.

[12] Zhang Y, Luo D, Li J, Li J (2021). Study on collision detection and force feedback algorithm in virtual surgery. J Healthc Eng.

[13] Kwon K, Park JS, Shin BS (2020). Virtual anatomical and endoscopic exploration method of internal human body for training simulator. J Korean Med Sci.

[14] Lee GK, Moshrefi S, Fuertes V, Veeravagu L, Nazerali R, Lin SJ (2020). What is your reality? virtual, augmented, and mixed reality in plastic surgery training, education, and practice. Plast Reconstruct Surg.

[15] Rose T, Nam CS, Chen KB (2018). Immersion of virtual reality for rehabilitation—review. Appl Ergon.

[16] Geng W, Du Y, Jin W, Wei W, Hu Y, Li J (2016). Gesture recognition by instantaneous surface EMG images. Sci Rep.

[17] Olsson P, Nysjo F, Hirsch JM, Carlbom IB (2013). A haptics-assisted cranio-maxillofacial surgery planning system for restoring skeletal anatomy in complex trauma cases. Int J Comput Assist Radiol Surg.

[18] Gratzel C, Fong T, Grange S, Baur C (2004). A non-contact mouse for surgeon-computer interaction. Technol Health Care.

[19] Ayoub A, Pulijala Y (2019). The application of virtual reality and augmented reality in oral and maxillofacial surgery. BMC Oral Health.

[20] Cheng M, Zhuang Y, Zhao H, Li M, Fan L, Yu H (2022). Development of a maxillofacial virtual surgical system based on biomechanical parameters of facial soft tissue. Int J Comput Assist Radiol Surg.

[21] Manek AS, Shenoy PD, Mohan MC, Venugopal WIS (2017). Aspect term extraction for sentiment analysis in large movie reviews using Gini Index feature selection method and SVM classifier. World Wide Web.

[22] Vaughan N, Dubey VN, Wainwright TW, Middleton RG (2016). A review of virtual reality based training simulators for orthopaedic surgery. Med Eng Phys.

[23] Akhtar K, Sugand K, Sperrin M, Cobb J, Standfield N, Gupte C (2015). Training safer orthopedic surgeons. Construct validation of a virtual-reality simulator for hip fracture surgery. Acta Orthop.

[24] Monteiro P, Goncalves G, Coelho H, Melo M, Bessa M (2021). Hands-free interaction in immersive virtual reality: a systematic review. IEEE Trans Vis Comput Graph.

[25] LeBlanc J, Hutchison C, Hu Y, Donnon T (2013). A comparison of orthopaedic resident performance on surgical fixation of an ulnar fracture using virtual reality and synthetic models. J Bone Joint Surg Am.

[26] Middleton RM, Alvand A, Garfjeld Roberts P, Hargrove C, Kirby G, Rees JL (2017). Simulation-based training platforms for arthroscopy: a randomized comparison of virtual reality learning to benchtop learning. Arthroscopy.

[27] Aim F, Lonjon G, Hannouche D, Nizard R (2016). Effectiveness of virtual reality training in orthopaedic surgery. Arthroscopy.

[28] Galvan-Ruiz J, Travieso-Gonzalez CM, Tejera-Fettmilch A, Pinan-Roescher A, Esteban-Hernandez L, Dominguez-Quintana L (2020). Perspective and evolution of gesture recognition for sign language: a review. Sensors (Basel).

[29] Zengeler N, Kopinski T, Handmann U (2018). Hand gesture recognition in automotive human(-)machine interaction using depth cameras. Sensors (Basel).

[30] van Amsterdam B, Clarkson MJ, Stoyanov D (2021). Gesture recognition in robotic surgery: a review. IEEE Trans Biomed Eng.

[31] Park BJ, Jang T, Choi JW, Kim N (2016). Gesture-controlled interface for contactless control of various computer programs with a hooking-based keyboard and mouse-mapping technique in the operating room. Comput Math Methods Med.

[32] Ruppert GC, Reis LO, Amorim PH, de Moraes TF, da Silva JV (2012). Touchless gesture user interface for interactive image visualization in urological surgery. World J Urol.

[33] Mewes A, Saalfeld P, Riabikin O, Skalej M, Hansen C (2016). A gesture-controlled projection display for CT-guided interventions. Int J Comput Assist Radiol Surg.

[34] Zou YB, Chen YM, Gao MK, Liu Q, Jiang SY, Lu JH, Huang C, Li ZY, Zhang DH (2017). Coronary heart disease preoperative gesture interactive diagnostic system based on augmented reality. J Med Syst.

[35] Qin C, Ran X, Wu Y, Chen X (2019). The development of non-contact user interface of a surgical navigation system based on multi-LSTM and a phantom experiment for zygomatic implant placement. Int J Comput Assist Radiol Surg.

[36] Forestier G, Petitjean F, Senin P, Despinoy F, Huaulme A, Fawaz HI, Weber J, Idoumghar L, Muller PA, Jannin P (2018). Surgical motion analysis using discriminative interpretable patterns. Artif Intell Med.

[37] Despinoy F, Bouget D, Forestier G, Penet C, Zemiti N, Poignet P, Jannin P (2016). Unsupervised trajectory segmentation for surgical gesture recognition in robotic training. IEEE Trans Biomed Eng.

[38] Pulijala Y, Ma M, Pears M, Peebles D, Ayoub A (2018). Effectiveness of immersive virtual reality in surgical training-a randomized control trial. J Oral Maxillofac Surg.

[39] Pugeault N, Bowden R (2011) Spelling it out: real-time asl fingerspelling recognition. In: 2011 IEEE international conference on computer vision workshops (ICCV workshops), pp 1114–1119

[40] De Smedt Q, Wannous H, Vandeborre J-P (2016). Skeleton-based dynamic hand gesture recognition. IEEE Conf Comput Vis Pattern Recognit Works CVPRW.

[41] Dong C, Leu MC, Yin ZZ (2015) Ieee, American sign language alphabet recognition using microsoft Kinect. In: IEEE conference on computer vision and pattern recognition (CVPR), Boston

[42] Ikram A, Liu Y (2021) Real time hand gesture recognition using leap motion controller based on CNN-SVM architechture. In: 2021 IEEE 7th international conference on virtual reality (ICVR), pp 5–9

[43] Loukas C, Rouseas C, Georgiou E (2013). The role of hand motion connectivity in the performance of laparoscopic procedures on a virtual reality simulator. Med Biol Eng Comput.

[44] Allgaier M, Chheang V, Saalfeld P, Apilla V, Huber T, Huettl F, Neyazi B, Sandalcioglu IE, Hansen C, Preim B, Saalfeld S (2022). A comparison of input devices for precise interaction tasks in VR-based surgical planning and training. Comput Biol Med.

[45] Pham D-M, Stuerzlinger W (2019) Is the pen mightier than the controller? A comparison of input devices for selection in virtual and augmented reality. In: 25th ACM symposium on virtual reality software and technology, pp 1–11

[46] Cercenelli L, Babini F, Badiali G, Battaglia S, Tarsitano A, Marchetti C, Marcelli E (2021). Augmented reality to assist skin paddle harvesting in osteomyocutaneous fibular flap reconstructive surgery: a pilot evaluation on a 3D-printed leg phantom. Front Oncol.

[47] Thabit A, Benmahdjoub M, van Veelen MC, Niessen WJ, Wolvius EB, van Walsum T (2022). Augmented reality navigation for minimally invasive craniosynostosis surgery: a phantom study. Int J Comput Assist Radiol Surg.

[48] Pose-Diez-de-la-Lastra A, Moreta-Martinez R, Garcia-Sevilla M, Garcia-Mato D, Calvo-Haro JA, Mediavilla-Santos L, Perez-Mananes R, von Haxthausen F, Pascau J (2022). HoloLens 1 vs HoloLens 2: improvements in the new model for orthopedic oncological interventions. Sensors (Basel).

